# Tobacco Control Measures' Role in Improving Employees' Health Under the Impact of Health Education and Media Campaign

**DOI:** 10.3389/fpubh.2022.904894

**Published:** 2022-07-07

**Authors:** Yu Wu

**Affiliations:** Media and Communication College, Weifang University, Weifang, China

**Keywords:** health education, media campaign, peer counselor training, tobacco control, employee health

## Abstract

The overarching purpose of this study was to investigate the impact of health education, media campaigns, and peer counselor training on employees' health. This study also attempted to evaluate the function of tobacco control as a mediator in the relationship between employees' health and health education, media campaigns, and peer counselor training. Data were collected from 440 tobacco industry workers in China using a questionnaire technique. Smart-PLS software and a structural equation modeling (SEM) technique were used to evaluate the data. Employees' health was found to be significantly improved by health education, media campaigns, and peer counselor training. Furthermore, tobacco control was discovered to moderate the association between employee health and health education, media campaigns, and peer counselor training. By analyzing the impact of health education, media campaigns, and peer counselor training on employees' health, this research provided an important theoretical contribution. In terms of practical applications, this study would help employees consuming tobacco to maintain a healthy and safe atmosphere that encourages them to be engaged and perform well. Furthermore, this study could prove effective in resolving difficulties linked to controlling employee tobacco addiction and improving their performance. The tiny sample size of this study, which included solely employees working in the Chinese tobacco sector, was one of its limitations. In addition, future studies can incorporate other constructs to acquire a deeper knowledge of the factors that influence employees' health.

## Introduction

Tobacco smoking represents one of the major global sources of disability and premature death decades after its consequences on human health. In 2017, smoking caused 7.1 million deaths globally, accounting for 7.3% of disability-adjusted years of life (1). Tobacco usage has negative economic consequences, such as decreased performance and increased healthcare expenditures (2, 3). These implications emphasize the significance of bolstering tobacco control, which is an important and critical step in governments' efforts to meet the goals for 2030 sustainable development (4). Tobacco usage is a key concern of the twenty-first century since tobacco-related mortality has been on the rise, decimating the youth and posing a threat to the environment. Smoking tobacco has claimed the deaths of 100 million individuals globally in the twentieth century, more than the combined deaths of World Wars I and II (5).

Cigarette smoking-related deaths would reach 1 billion in the twenty-first century if the prevalence of tobacco consumption patterns continues (6). In the later part of the twentieth century, a very lucrative sector fostered the tobacco menace by promoting an extremely addictive commodity while taking advantage of globalization. Authorities and public health agencies realized the impact globalization of tobacco had on the effects of smoking and its progression into a huge epidemic (7). Tobacco's substantial economic cost to society, which now totals USD $1,436 billion or 1.8% of global yearly GDP, was quickly understood (5). Simultaneously, policymakers and public health agencies realized that the epidemic required a worldwide, collective approach at the highest level. In 1999, the World Health Organization (WHO) began the process of establishing the Framework Convention on Tobacco Control, the very first international agreement under WHO's supervision. As a result, the world community began to identify tobacco smoking as a serious threat to human health and economic and social issues and to take collective international action (8).

The “Framework Convention on Tobacco Control,” a first international health agreement aimed at bolstering tobacco consumption reduction efforts across participating member countries, was developed by WHO in 2003. In 2008, the WHO produced a package based on acronyms reflecting six facts-based control approaches to help countries execute tobacco control policies. Although tobacco control agencies have advanced the implementation of certain demanding reduction strategies in the last 10 years, there are still numerous obstacles to overcome in order to reduce population-level tobacco consumption (9).

Despite the diverse stages of the tobacco pandemic and tobacco management in different countries, it is vital to consolidate the knowledge base on the efficacy of strategies in reducing smoking as governments consider how to improve. Organizations are the players which contribute to the national economy; therefore, there was a need to emphasize the role of tobacco use and its control measures among employees in such working setups. Despite improved job flexibility in a variety of professions and businesses, numerous individuals find themselves spending considerable periods of time on the job or doing employment activities on the job. Therefore, as a result, the workplace is a perfect place for businesses to address their employees' general health and wellbeing (10).

Whereas, many workplace treatments have typically concentrated on overall health, the national trend continues to shift toward a more holistic approach to overall health that attempts to preserve a mix of physical, cognitive, and lifestyle issues. Seemingly, companies are now more interested in the relationship between the personal health of employees and workplace efficiency, work performance, medical costs, absence, and presence (11). Any business can suffer financial and productivity losses as a result of illness or injury. Businesses and organizations are seeking ways to save costs while maintaining high efficiency. Many firms are implementing health wellness programs that are encouraged at the company level in order to encourage improved health among their employees (11). Employees spend a significant amount of time at work each day, making it an ideal venue for strategies for addressing controllable behavioral health disparities. Previous analyses of workplace-based controlled trials suggest how they can be helpful in altering a variety of critical variables such as nutrition, activity level, obesity, hazardous alcohol consumption, and tobacco use.

Unfortunately, workplace interventions are frequently under-implemented, reducing their effect on employee health. Implementing strategies such as tobacco control measures might be beneficial to employees' health. Improving the benefits of health programs has previously been shown to be quite successful in reducing the incidence of severe heart disease and diabetes studies (12). Is there a link between health education and the good health of employees? An increasing number of studies have been looking into the impact of health education on employees' health. Sometimes in highly advanced regions, such as the United States, individuals with lower levels of education have been found to have poorer health than other populations. These huge health inequities caused by education are thought to be the cause of this pattern (13). A good grasp of health education's advantages can thus be the key to lowering health inequities and increasing future individuals' health. Despite the increased interest, studies in the education–health field has yet to provide definitive answers to certain key questions. Our study, therefore, tried to fill this gap by evaluating the impact of health education as a tobacco control measure in the context of employees' health at work. While the link involving health education and employees' health has been proven, the reasons for this link have yet to be discovered (14).

Employees with a high degree of education have better health, as evidenced by their high self-reported health and reduced rates of illness, death, and disability. To reach a big audience, media campaigns employ television broadcasts, newspapers, the Internet or social networking sites, radio transmissions, or other manifestations.

Tobacco-specific advertising informs existing and potential tobacco users on the risks of smoking, and they frequently include graphic depictions or emotive messaging to impact beliefs and attitudes about tobacco use (15). There is substantial evidence showing that mainstream media campaigns decrease tobacco consumption, improve cessation outcomes, and minimize youth cigarette consumption. Elevated, well-funded TV campaigns and initiatives that are components of a complete tobacco control program have the best data. Adult and youth tobacco use are reduced as a result of media campaigns, as is the commencement of tobacco use by young people. Campaigns like these have been shown to decrease tobacco usage, enhance quit rates, and improve the use of cessation facilities. Light smokers may benefit more from campaigns than daily smokers. None of these researchers focused on campaigns at an organizational level as tobacco control measures for the employees, leaving a gap in organizational management, so we tried to find the impact of media campaigns on improving employees' health. Along with health education and media campaigns, peer counselor training could also provide great impact as a tobacco control measure at work. It could also improve the general health of employees; therefore, we utilized this concept as well for evaluating the outcomes. Peer counselor training can help in modifying the behavioral orientation of employees toward tobacco consumption and help in overcoming the addiction to tobacco use (16). This study tried to answer certain questions raised in organizational management by evaluating the role of health education, media campaigns, and peer counselor training in improving employees' health. The mediating role of tobacco control measures was also analyzed in this research.

## Theoretical Support and Hypothesis Development

This research gets support from the theory of planned behaviors (TPB), which target the tobacco control measures for the employees, who in return receive improved health. Icek Ajzen established the (TPB) in an effort to determine human behavior (17). TPB is an approach to identifying behavioral, ethical, and control factors which influence behavior. Strategies can then be devised to address and modify such concepts or the emphasis placed on these, resulting in changes in mindset, behavioral control, or control beliefs, as well as intents and practices. Exercising, tobacco and drug use, disease preventive behaviors, and other health behaviors have all been correctly predicted and explained using the TPB (16).

Modeling of behavior, such as the TPB, therefore provides a theoretical strategy that allows program designers and planners to identify the foundational pillars that drive behavior and, as a result, construct appropriate approaches. This study also got support from the rational model given by a previous study (18), also known as the KAP model, which is derived from knowledge, attitude, and practice. This model supports the education of health among the individuals, which impacts the health of people. According to this model, knowledge can be obtained, retained, and utilized for the betterment. This can influence the attitudes and practices of individuals, which could lead to improved health. In this regard, some studies reported that people were not aware of the hazards of tobacco which lead to compromised health (19) so educating them could have improved their health.

Health education is a helpful tool in spreading the awareness among people and is modifying the intentions and behaviors of people specifically for the improvement of health and is supported by several other theories. These theories which provide a basis for health education include several models such as the trans-theoretical model of change (20), the health belief model (21), the extended parallel process model (22), the activated health education model (23), the social cognitive theory (24), and the diffusion of innovation theory (25). These theories and models provide an understanding of the role of health education for the individual (employees in this study) (26).

### Association of Health Education With Employee Health

An important social factor of health is health education (or absence thereof). Although there are many multiple definitions of health education, they all reflect “the abilities that enable people to access, interpret, assess, as well as using knowledge to take the right steps which have an effect on their general health” (27). Decreased ranks of health education have been linked to more hospitalizations, more emergency room visits, a poorer ability to demonstrate proper medication administration, a poorer ability to comprehend labels and health messages, a relatively poor overall wellness, and higher death rates in the general population (28). Lower levels of health education are connected with less optimism in lifestyle modifications, less proactive in health-related behaviors, avoiding proper healthcare, rejection of health issues, and lower quality of life (e.g., death rates, hospitalization) in individuals with chronic conditions compared to peers having higher levels of health literacy. Health education is not a company's fixed asset; this can be upgraded through various health programs and is tailored toward both context and meaning (27). Previous research suggests that interventions enhance healthcare education, as well as proxies indicators of information, mindset, and behavior (27). Furthermore, health education promotion may not have a direct impact on health outcomes; customizing behavioral treatments to one's health status may reduce the effect of such approaches (28). As a result, it is possible to claim that promoting health education is a key paradigm for combating the rising burden of non-communicable diseases, particularly ones linked to health behaviors. All these significant evidence about health education for the improvement of the health of individuals suggest that, if attention is focused on educating the employees about tobacco control, then it could lead to improvement in their health. Therefore, the author devised the following hypothesis.

*H*_1_. *Health education has a positive and significant impact on employee health*

### Media Campaign and Employee Health

Media campaigns have long been utilized as a tool for public health promotion, and their success has been evaluated and documented in a variety of publications (29). Studies concentrating on the implementation of new behaviors rather than the avoidance or discontinuation of problem behaviors, as well as those with concurrent law enforcement features, have been connected to successful campaigns. Figures show that campaigns concentrating on tobacco and alcohol consumption are more effective than those concentrating on illegal drugs when it comes to media campaigns designed to prevent or reduce narcotic usage (28).

Campaigns in the media can last for a short time or for a long time. These can operate independently or be connected with other structured program components, including medical or organizational engagement and simple exposure to technology or current goods or services. These can be used to supplement changes in policy. Whenever health campaigns become part of bigger societal marketing campaigns, other techniques of transmission may be used (28). To alter the behavior of entire populations, mass media campaigns can use both direct and indirect paths. Various campaigns try to have a direct impact on individuals by eliciting emotional or cognitive responses (30).

Individual decision-making processes are meant to be influenced by such programs. An elimination or reduction of barriers to healthy transformation, supporting people in adopting healthy or recognizing harmful social norms, and associating desired feelings with change are all expected consequences. These modifications boost the chances of acquiring new behaviors by strengthening intentions to change (30). There has been far more research on the impact of media campaigns on smoking than on any other wellness topic, and the support for the benefit is substantial as a result (28). It is well-understood that media campaigns can help in eliminating negative health behaviors such as tobacco consumption; therefore, it suggests the beneficial role of media campaigns in organizations to improve employees' health. The following hypothesis was developed in this connection.

*H*_2_. *Media campaign significantly affects employees' health*

### Peer Counselor Training and Employees' Health

“A personal service offered by an individual with a major illness to an individual with a serious disorder,” according to the definition of peer support (31). Such specialized help provides support networks prior to, throughout, and after therapy to aid good recovery in the healing person's group. Peer support is included in a larger rehabilitation strategy that prioritizes person-centered outcomes like social integration and independence over conventional health outcomes like neuropsychiatric manifestations. Emotional and practical assistance, good self, encouraging optimism, empowerment, self-efficacy, and increasing social networks are all ways that peers can aid their own and others' recovery (30). Peers can also provide a variety of services, such as social support, illness care, counseling, communication, training, and advocating, which are formalized in positions such as peer companions, peer advocacy, client case managers, peer professionals, or peer counselors (32). Peer assistance can be delivered in a variety of contexts as a standalone service or as an integrated aspect of professional care. As a result, it creates opportunities for people who have experience with mental illness to connect with those who are in desperate need of help but are typically excluded from standard health services (33). In high-income countries, both quantitative and qualitative research have proved the far-reaching effect of peer support. Independence, optimism, healthy life, self-esteem, social integration, and participation in the care of the patients are all positive outcomes (30). Along with this, peer counselor training has also proved its worth in improving the health of people (34). Therefore, keeping this in view, we devised the following hypothesis suggesting that peer counselor training can also improve employees' health.

*H*_3_. *Peer counselor training has a significant impact on employee health*

### Mediating Role of Tobacco Controls

Tobacco control measures in developed countries have shown a lot of success while in developing countries, there has been a lack of implementation of such tobacco control measures. There could be strict monitoring for the implementation of these measures in controlled environments such as workplaces. Cigarette excise fees and fresh indoor air legislation, among other tobacco control programs, have worked to minimize cigarette smoking rates in the United States (30). On a national and federal level, however, these rules differ significantly. There are 633 municipal governments in the United States that all had their own tobacco taxation rates, ranging from $1.25 to 7.16 per pack as of March 2021 (35, 36). Local clean indoor air regulations differ as well, particularly for areas in states without nationwide clean indoor air legislation. Local diversity in tobacco control policies must therefore be taken into account in research on the influence of tobacco control measures on tobacco cessation (37). Furthermore, smoke reduction efforts may not benefit all population groupings equally (38). Despite the implementation of these regulations, low-income smokers' attempts to quit are less effective despite the fact that individuals are just as inclined to try as other smokers (39, 40).

Many low-income smokers report bad past quitting experiences and internalized tobacco guilt, which may be linked to lower self-efficacy for stopping. Smokers from low socioeconomic class suffer additional barriers to quitting, including higher addiction to nicotine and smoker-dominated social networks, such as high pro-smoking social standards (30). Such tobacco control measures and their influences suggested that they could provide a mediating role mentioned in the following hypothesis.

*H*_4_. *Tobacco control plays a mediating role between health education and employees' health*
*H*_5_. *Tobacco control plays a mediating role between media campaigns and employees' health*
*H*_6_. *The relationship between peer counselor training and employees' health is mediated by tobacco controls*

**Figure 1:**
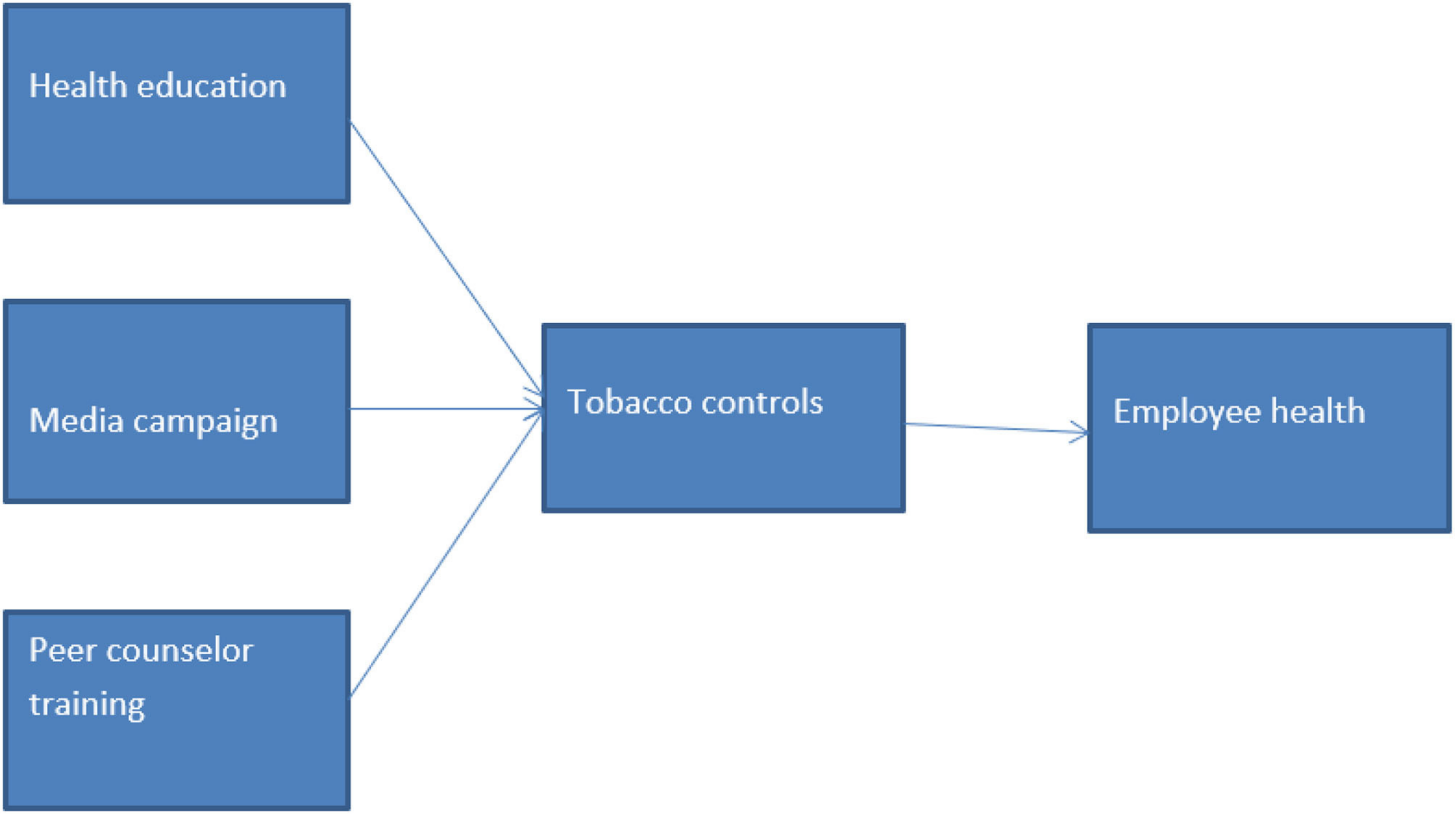
Conceptual framework.

Based on the literature and the hypotheses, the following framework has been established as shown in Figure 1.

## Methodology

The current study used a quantitative method with a deductive approach, in which hypotheses were developed and tested to discover how specific factors affected other variables. Researchers utilize this practice to ensure that there is no bias. To collect data for this quantitative study's analysis, self-administered surveys were used. The study's target group was the employee working in the Chinese tobacco industry. In this study, the convenience sample approach was employed to obtain data from respondents *via* physically distributed questionnaires. The data collection process took 2–3 months to complete. The total number of questionnaires distributed was ~600, and we obtained 410 responses, resulting in a 73% response rate; of those, 440 responses were viable and used for data analysis. Because the data collection technique was initially delayed, reminders were sent to selected respondents to expedite the process. Our study unit of analysis was Chinese employees working in the Tobacco industry.

### Data Analysis Technique

In this study, Smart-PLS 3 was utilized to evaluate the data. In our research, the structural equation modeling was applied. Partial least square is frequently utilized in management and social sciences since it is a variance-based structural equation modeling technique (41). PLS-SEM is also a causal modeling technique whose purpose is to increase the explained variance of latent dependent components. Researchers regard PLS-SEM as a “magic bullet” for dealing with empirical findings with a limited sample size (42). Smart-PLS is easy to use and contains a wealth of complex capabilities (43). Furthermore, the Smart-PLS approach is best suited to complex equation studies (44). This study follows the suggestions of a previous study (44) to properly calculate the values of beta, reliability, and standard error and ensures that all of these indicators are part of their respective latent variables with outer loadings of 0.7 in the reflecting outer model evaluation.

### Measurement

In this study, a 5-point Likert scale ranging from strongly agree to strongly disagree was employed to record respondents' responses. When assessing the dependability of each variable, the Cronbach alpha value should be larger than 0.70 (45). We have given the measurement as well as the Cronbach alpha:

#### Health Education

This study used the scale of Brakel et al. (46) which consists of 18 items. The Cronbach alpha value is 0.947, which is acceptable when compared with the benchmark value.

#### Media Campaigns

The current study adopted the 12-items scale of Mirbagheri (47), and the Cronbach alpha value is 0.955, which is far above the required value.

#### Peer Counselor Training

Peer counselor training was measured by using the scale of Berkmal et al. (48). The Cronbach alpha value of peer counselor training is 0.932, which is acceptable.

#### Tobacco Control

The current study adapted the scale of Rahman et al. (49) which consists of 3 items. The Cronbach alpha value is 0.897, which is acceptable.

#### Employee Health

Employee health was measured by utilizing the scale of Becker et al. (50) consisting of 18 items. The Cronbach alpha value is 0.947, which is acceptable.

### Demographic Analysis

Table 1 displays the demographic information of the respondents who took part in the study. Our overall number of acceptable responses was 440, with 312 men and 128 women. A total of 14.5% of the respondents were between the ages of 25 and 35, 37.8% were between the ages of 35 and 45, 42% were between the ages of 45 and 55, and 5.7% were over 55. Bachelor's degree holders made up 68.65% of all participants, followed by master's degree holders (31.35%). Furthermore, employees with less than a year of organizational tenure made up 26.20%, employees with 1–3 years made up 40.63%, employees with 4–6 years made up 27.98%, and employees with more than 6 years made up 5.19%.

**Table 1:** Demographic details.

**Variable**	**Groups**	**Percentage**	**Total**
Gender	Male	71%	440
	Female	29%	
Education	Graduation	68%	440
	Masters	32%	
Age	25–35	14.5%	
	35–45	37.8%	
	45–55	42%	
	Above 55	5.7%	
Experience	<1 year	26.20%	
	1–3 years	40.63%	
	4–6 years	27.98%	
	More than 6	5.19%	

### Common Method Bias

Table 2 explains the most common method bias, which is the bias of the questionnaire. The percentage of variance for a single item must be <50% (51). Bias is not present in the data because the outcome for the total variance explained in the current study is <50%.

**Table 2:** Common method bias.

**Component**	**Initial eigenvalues**			**Extraction sums of squared loadings**		
	**Total**	**% of variance**	**Cumulative %**	**Total**	**% of variance**	**Cumulative %**
1	12.764	53.185	53.185	12.764	42.440	53.185
2	2.047	8.531	61.716			
3	0.878	3.657	65.373			
4	0.786	3.276	68.648			
5	0.713	2.970	71.618			
6	0.701	2.921	74.539			
7	0.624	2.600	77.138			
8	0.502	2.091	79.230			
9	0.463	1.928	81.158			
10	0.451	1.878	83.035			
11	0.403	1.680	84.716			
12	0.394	1.642	86.358			
13	0.374	1.558	87.916			
14	0.355	1.479	89.395			
15	0.332	1.384	90.779			
16	0.323	1.345	92.124			
17	0.314	1.309	93.433			
18	0.285	1.189	94.622			
19	0.256	1.068	95.690			
20	0.248	1.032	96.721			
21	0.225	0.937	97.658			
22	0.201	0.839	98.497			
23	0.194	0.808	99.305			
24	0.167	0.695	100.000			

*Extraction method: principal component analysis*.

## Data Analysis and Results

### Measurement Model

**Figure 2:**
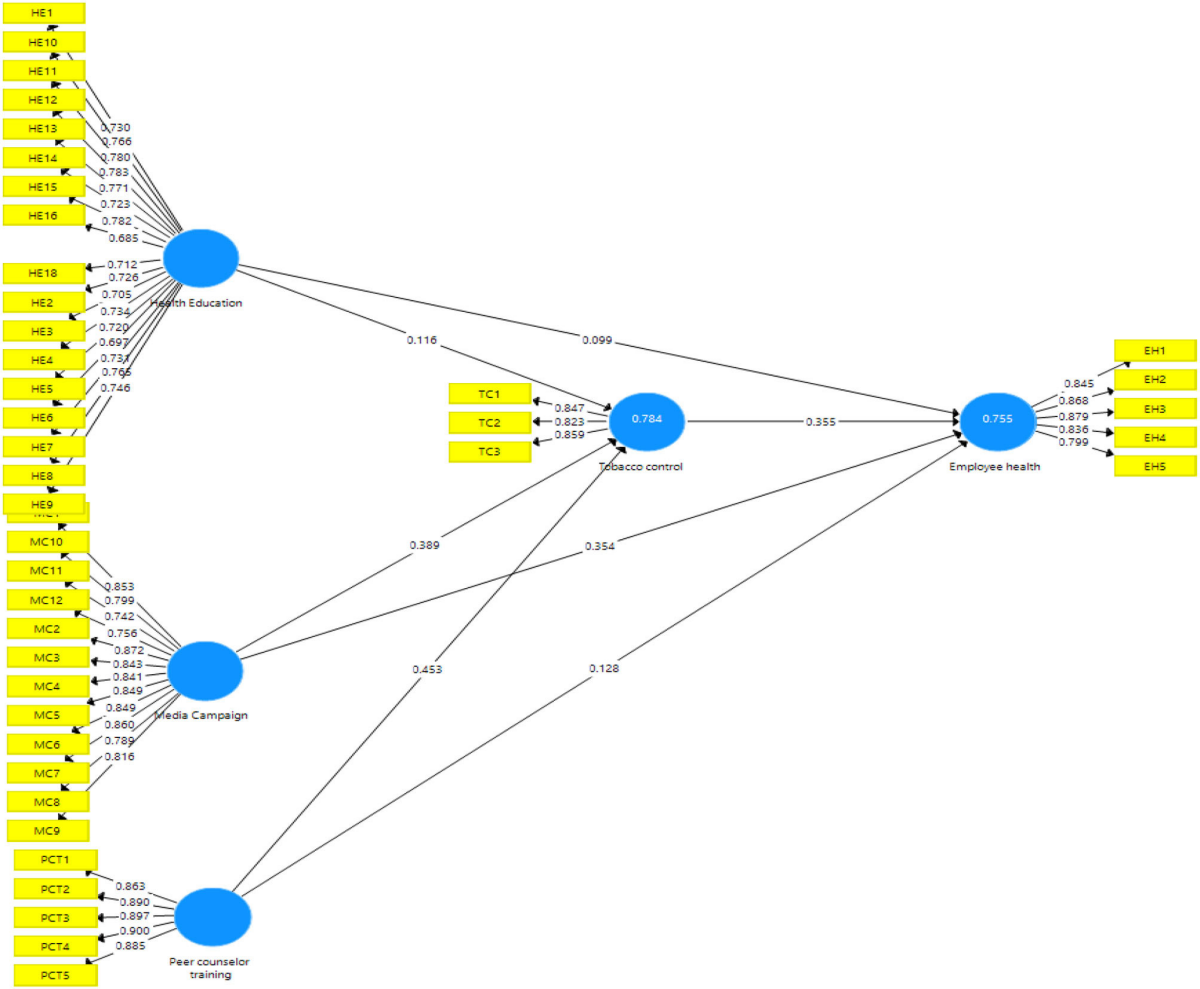
Measurement analysis.

The output measurement model's algorithm is represented in Figure 2. This diagram depicts the effect of independent variables on the dependent variables of the study.

Table 3 illustrates the factor loadings for each research construct, which are academic self-efficacy, academic motivation, learning agility, student sustained engagement, and student academic accomplishment. In addition to the VIF values, the table shows the extracted composite reliability and average variance (AVE). The factor loading describes an item's contribution to the variable and must be >0.60 (52). All of the factor loadings in this study are more than 0.60, indicating that the factor loadings are fair. The model's collinearity issues are validated by the variation inflation factor (VIF). The current study's outer VIF result is also <5 (range from 1.804 to 4.211), showing that the model is not collinear. Furthermore, the present inner VIF result is <5 (within 1.508–2.475; between 1.508 and 2.475). Table 3 demonstrates that the AVE values are more than 0.60, indicating the presence of convergent validity. The composite reliability was better than 0.70, putting it in the highly satisfactory category.

**Table 3:** Factor loadings–Cronbach alpha, composite reliability, and AVE.

**Constructs**	**Items**	**FL**	**VIF**	**Cronbach's alpha**	**CR**	**AVE**
Employee health				0.897	0.922	0.664
	EH1	0.837	2.289			
	EH2	0.858	2.69			
	EH3	0.873	2.926			
	EH4	0.828	2.277			
	EH5	0.797	1.978			
	EH6	0.682	1.521			
Health education				0.947	0.952	0.54
	HE1	0.735	2.792			
	HE2	0.725	2.847			
	HE3	0.713	2.503			
	HE4	0.737	2.526			
	HE5	0.721	2.311			
	HE6	0.693	2.013			
	HE7	0.727	2.199			
	HE8	0.753	2.509			
	HE10	0.765	2.539			
	HE11	0.784	2.811			
	HE12	0.779	2.78			
	HE13	0.768	2.34			
	HE14	0.723	2.13			
	HE15	0.784	2.41			
	HE16	0.687	2.012			
	HE17	0.663	1.867			
	HE18	0.721	2.15			
Tobacco control				0.797	0.881	0.711
	TC1	0.846	1.744			
	TC2	0.823	1.624			
	TC3	0.86	1.732			
Media Campaign				0.955	0.961	0.713
	MC1	0.863	3.609			
	MC2	0.883	4.199			
	MC3	0.847	2.951			
	MC4	0.854	3.093			
	MC5	0.863	3.584			
	MC6	0.863	3.464			
	MC7	0.872	3.554			
	MC8	0.801	2.435			
	MC9	0.819	2.964			
	MC10	0.769	2.493			
Peer counselor training				0.932	0.949	0.787
	PCT1	0.863	2.641			
	PCT2	0.89	3.173			
	PCT3	0.897	3.423			
	PCT4	0.9	3.424			
	PCT5	0.884	2.99			

*AVE, Average variance extracted; CR, Composite reliability; EH, Employee health; HE, Health education; ISQ, Tobacco control; MC, Media campaign; PCT, Peer counselor training*.

To examine discriminant validity, the HTMT ratio and the Fornell and Larcker Criteria were utilized (see Tables 4, 5). These tests examine whether or not a difference exists between the variables. The HTMT ratio should be <0.90 to ensure the discriminant validity of a variable (7). The current study's HTMT ratio was smaller than 0.90, showing that discriminant validity existed. According to the Fornell and Larcker Criteria, the value at the top of the column must be greater than the value -given in column (7) of Table 5.

**Table 4:** Discriminant validity (HTMT ratio).

	**EH**	**HE**	**TC**	**MC**	**PCT**
EH	0.852				
HE	0.717	0.81			
TC	0.826	0.729	0.843		
MC	0.814	0.762	0.812	0.844	
PCT	0.764	0.686	0.814	0.752	0.887

*EH, Employee health; HE, Health education; TC, Tobacco control; MC, Media campaign; PCT, Peer counselor training*.

**Table 5:** Discriminant validity (Fornell and Larcker Criteria).

	**EH**	**HE**	**TC**	**MC**	**PCT**
EH					
HE	0.765				
TC	0.826	0.825			
MC	0.814	0.791	0.798		
PCT	0.832	0.718	0.811	0.797	

*EH, Employee health; HE, Health education; TC, Tobacco control; MC, Media campaign; PCT, Peer counselor training*.

An *R*-square score of 0.50 or higher implies that the model is substantial and good (53). The R-square values for the variables in the current study are close to or more than 0.50, indicating that the model is adequate (see Table 6). As measured by Q-square, cross-validated redundancy should be greater than zero (7). The Q-square values for the variables in the current study are more than zero, indicating that the model is significant.

**Table 6:** *R*-Square values and *Q*-Square values for the variables.

	***R*** **^2^**	**Q^2^**
Employee health	0.753	0.494
Tobacco control	0.78	**0.494**

*Bold indicates the relationship value for the variables*.

### Structural Model Assessment

**Figure 3:**
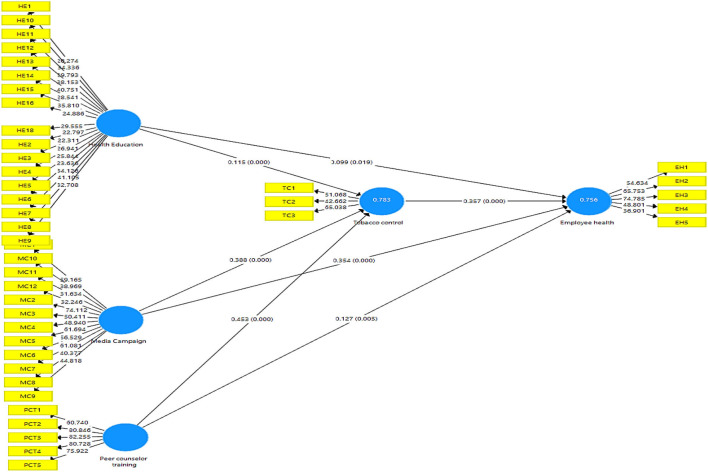
Structural analysis.

In the next step, a structural model was evaluated after the establishment of the measurement model of the constructs (see Figure 3).

Bootstrapping technique of 5,000 samples was run in Smart-PLS for assessment of the structural model. In the structural assessment model, we analyzed the coefficient of determination (*R*^2^), predictive relevance, significance, and relevance of the path coefficient. *R*-Square statistics reflect how much change in an endogenous variable happened because of an exogenous variable. The *R*^2^ value of 0.75, 0.50, or 0.25 is considered “substantial, moderate, and weak” (54), respectively. In this study, *R*^2^ values of endogenous constructs, i.e., employee health and tobacco control are 0.753 and 0.781, respectively, as shown in Table 6, which is considered substantial as per the criteria. Q-square indicates predictive relevance and measures whether a model has predictive relevance or not (>0 is good). In this study, Q-square values are >0, which reflects the predictive relevance of the model as shown in Table 6.

In the next step, the hypotheses of the study were evaluated by analyzing the significance and relevance of the path coefficient. The bootstrapping procedure was applied by using 5,000 bootstrap samples for the assessment of the path coefficient.

H1 proposed that health education has positive and significant impacts on employee health. The results illustrate that health education has a significant and positive impact on employees' health (B = 0.100, *T*-value = 2.419, *p* = 0.016). Thus, H1 is accepted. H2 proposes a media campaign that has positive and significant impacts on employees' health. The result is in alignment with what we proposed, that is, media campaigns significantly and positively affects employee health (B = 0.339, *T*-value= 6.740, *p* = 0.000). As the *p*-value is <0.05, H2 is also accepted. H3 results show that peer counselor training has a positive and significant impact on employees' health (B = 0.137, *T*-value = 3.085, *p* = 0.002). Hence, H3 was accepted (see Table 7).

**Table 7:** Direct effect.

**Hypotheses**	**Relationship**	**Beta**	**SD**	***T*** **-value**	***P*** **-values**	**Confidence interval**		**Decision**
						**LL**	**UL**	
H1-	Health Education -> Employee health	0.100	0.041	2.419	0.016	0.019	0.174	Supported
H2	Media Campaign -> Employee health	0.339	0.050	6.740	0.000	0.239	0.436	Supported
H3	Peer counselor training -> Employee health	0.137	0.044	3.085	0.002	0.049	0.228	Supported

Mediation analysis was performed by analyzing the total, direct, and indirect effects. In this study, three mediation hypotheses were proposed. In this study, mediation analysis was performed by using the method described in an earlier study (55). H4 results show that tobacco control partially mediates the relationship between health education and employee health as both direct and indirect effect are significant (B = 0.048, *p* = 0.000). Similarly, tobacco control partially mediates the relationship between media campaigns, employees' health (B = 0.128, *p* = 0.000), and peer counselor training and employee health (B = 0.175, *p* = 0.000) (Table 8).

**Table 8:** Indirect effects.

**Hypotheses**	**Constructs**	**Total effect**		**Direct effect**		**Indirect effect**		**Confidence interval**	
		**Beta**	***P*** **-value**	**Beta**	***P*** **-value**	**Beta**	***P*** **-value**	**LL**	**UL**
H4	HE -> TC -> EH	0.148	0.001	0.100	0.016	0.048	0.000	0.024	0.075
H5	MC -> TC -> EH	0.466	0.000	0.339	0.000	0.128	0.000	0.080	0.178
H6	PCT -> TC -> EH	0.312	0.000	0.137	0.002	0.175	0.000	0.118	0.231

## Discussion

Tobacco control measures have been implemented worldwide in various setups and have produced some significant contributions to improving the health of people. This study was conducted in the context of organizational management for the improvement of employees' health. The role of health education was assessed in improving employees' health. This study also provides insights into the role of media campaigns and peer counselor training in improving employees' health. Moreover, this research also looked into the mediating role of tobacco controls between health education, media campaigns, peer counselor training, and employees' health. The results showed the importance of research for the employees of tobacco-related organizations. It was evident from the previous research that smoking and tobacco consumption had been associated with several negative impacts on people globally and resulted in life losses and severe diseases (1).

It was also evident that the consumption of tobacco also had enormous impacts on the economic outcome of businesses, such as reduced performance of employees and increased burden on healthcare expenditures (2, 3). The direct impact of health education proved that, if employees are given proper education about their health improvement, then it provides improved health for employees within the workplace. These results are in accordance with some previous researchers who asserted on spending money on the healthcare of workers in the workplace (27). The previous research also suggested that such education on health improves healthcare and improves the mindsets and behaviors of workers as well (28, 56–58). These results are obtained due to the fact that people generally spend more time in their workplaces and do not usually have time for educating themselves at home or in their free time. The direct effects of media campaigns were also tested in this research and proved that such media campaigns at workplaces could help in improving employees' health. This result is also supported by the fact that people usually do not have enough time to spend other than in their workplaces for educating themselves about these things. Media campaigns have previously been shown to have strong effects on achieving the targets.

Media campaigns are being used as a tool for the promotion of public health, and their success has been evaluated and documented in a variety of studies (29). Similar kinds of results have been obtained in some of the studies, which looked into the role of media campaigns on the general health of people (59–61). The direct effects of peer counselor training also proved that it could improve employees' health at the workplace. The possible reason for such results lies in the fact that peers are those individuals who are working with them in the same setups. The only difference is that they have gone through tobacco consumption and have the experience to share with their colleagues and help them train to quit tobacco use or avoid it.

As peers are connected to their colleagues, peer assistance could be delivered in a mix of approaches as a sole service or as an integrated aspect of professional care (29). It has been proved that peer counselor training could help in improving the health of individuals (34). This research also focused on the mediating role of tobacco control measures. It is worth mentioning that, in previous years, countries have faced a lot of causalities and health impairments due to smoking and tobacco use. Millions of people have lost their lives in the United States alone (1). Thousands of deaths have been reported in the last decade due to health issues that arose from tobacco consumption, and it has produced many economic consequences for the public (29). This could have been avoided.

Many developed countries like the United States have regulated certain policies and measures to control tobacco use at large as well as at local and organizational levels. Different states have imposed certain restrictions like clean air taxes and cigarette taxes. This helped in reducing the impacts of tobacco use on the general public (29). It was also considered in the recent past that local diversity in tobacco control policies must therefore be taken into account in research on the influence of tobacco control measures on tobacco cessation (37). Such tobacco control measures could influence the relationships leading to improved health of people. Therefore, the results of the current study indicated that tobacco control provided a mediating impact among health education, media campaigns, peer counselor training, and employees' health. No previous investigations found such mediating role of tobacco control before. It aided and enhanced these relationships and provides a strong foundation for future research in this regard.

## Conclusion

There is a growing emphasis placed by researchers and practitioners regarding employee health and safety issues in current literature. Research and practitioners are showing their interest to explore the different predictors and antecedents of health issues among the employees from an organizational perspective. This study's main aim was to investigate the three key predictors of employee health, i.e., health education, media campaign, and peer counselor training on employee health. This study also explores the underlying mechanism of tobacco control to explore the impact of health education, media campaign, and peer counselor training on employee health. The data of this study were collected from the employees working in tobacco-producing company. The PLS_SEM technique was adopted for data analysis purposes. The finding reveals that health education, media campaign, and peer counselor training has positive and significant impacts on employees' health. In addition to this, tobacco control mediates the relationship. This study contributes to the body of literature by exploring the key predictors of employee health, which may be beneficial for researchers. This study provides valuable thoughts for policymakers and organization management to adopt the health culture in the organization, which ultimately impacts the overall organization's performance.

### Managerial and Theoretical Implications

The finding of this study reveals important predictors of tobacco control among employees, which may have positive impacts on employees' health. This study focused on the role of health education in tobacco control. The results show that health education plays a vital role in improving the employee's health. Hence, organizations should focus on the health of employees because healthy employees are more productive and perform better. In this context, the organization's management should conduct seminars and training sessions to increase awareness of health education among the employees. Similarly, massive media campaigns and peer counselor training also help to improve the employee's health, so the organization should use their internal media campaign to raise awareness among smokers regarding the negative consequences of smoking behaviors.

This study also reveals that tobacco control mediates the impacts of health education, media campaign, and peer counselor training on health education. Studies reveal that smoking/tobacco consumption increases among the employees due to several factors such as job burnout, mental depression, emotional exhaustion, and work-life conflicts; thus, this study provides valuable insights to policymakers for the realization of different important factors which facilitate to reduce the tobacco consumption among the employees. This study was cross-sectional by nature and investigated the key predictors which affect tobacco control. This study provides a causal path through which health education, media campaign, and peer counselor training impact employees' health. This study suggests that policymakers create mass awareness among the employees to discourage smoking behaviors.

Government and NGOs should conduct various seminars and training sessions to promote health education. There should be a proper mechanism in the organization to promote healthy behaviors because a healthy culture leads to an increase in the productivity of the staff. The organization's management should launch various incentive programs to encourage smokers to quit their smoking habits. The government should also implement and strengthen laws to discourage tobacco consumption. This study also has theoretical significance as this study investigates the important determinants and predictors of tobacco control and employee health from the employee perspective. This study also contributes to health research from an organizational perspective. Organizations should implement social policies to facilitate the employees to adopt a healthy lifestyle and also discourage tobacco consumption. This study also provides valuable directions to research scholars to explore the key factors which are affecting the health of employees. This study also explores the underlying mechanism through which key predictors impact employee health. Future studies should explore psychological factors as predictors and mediators of employee health.

### Limitation of the Study

Despite several important contributions, this study has some shortcomings that should be addressed in future studies to get better findings. First, the sample size of this study was limited, which may risk for generalization of the results, so future studies should increase the sample size for the assessment of key potential determinants of tobacco control. The current study collected data from China only, so future studies might collect data from multiple countries and conduct comparative analyses. Secondly, data of this study were collected from only a single-source, which may create potential issues of common method biases, so future studies should be collecting data from multiple sources to avoid this issue. Future should explore more key predictors of tobacco control and employee health.

## Data Availability Statement

The original contributions presented in the study are included in the article/supplementary material, further inquiries can be directed to the corresponding author/s.

## Author Contributions

YW has worked on the conceptual framework, data collection, and draft writing. She has agreed on the final version of the manuscript.

## Conflict of Interest

The author declares that the research was conducted in the absence of any commercial or financial relationships that could be construed as a potential conflict of interest.

## Publisher's Note

All claims expressed in this article are solely those of the authors and do not necessarily represent those of their affiliated organizations, or those of the publisher, the editors and the reviewers. Any product that may be evaluated in this article, or claim that may be made by its manufacturer, is not guaranteed or endorsed by the publisher.
